# Ought Craniotomy to Be Abolished?

**Published:** 1887-03

**Authors:** Joseph Griffiths Swayne

**Affiliations:** Consulting Physician Accoucheur to the Bristol General Hospital; Lecturer on Obstetric Medicine at the Bristol Medical School


THE BRISTOL

### Medico-Chirurgical Journal.

MARCH, 1887.

### OUGHT CRANIOTOMY TO BE ABOLISHED?

Read at the Bath and Bristol Branch of the

British Medical Association,

Jan. 26th, 1887.

BY

JOSEPH GRIFFITHS SWAYNE, M.D.,

*Consulting Physician Accoucheur to the Bristol General Hospital;*
*Lecturer on Obstetric Medicine at the Bristol Medical School.*

THE question whether craniotomy ought to be abolished has again come prominently to the front—chiefly in consequence of the brilliant successes which have recently been achieved in abdominal surgery. At the meeting of our Association in Brighton, last year, it was brought forward by Dr. Meadows, the president of the Obstetrical Section, and also by Dr. Barnes, who is, perhaps, the best authority in this country on operative midwifery; and it gave rise to much interesting discussion. But, in consequence of the restriction as to time, it was impossible for other speakers to give the full results of their experience in relation to this most important question. Since that time the subject has been again discussed, at a meeting of the Gynæcological Society in October last, and it gave rise to a long debate, which did not end, however, in any very definite conclusion. The question is so momentous that it cannot be too fully discussed, and I therefore feel that I shall not be unnecessarily occupying time this evening if I give reasons, based on my own experience, for the conclusions which I have arrived at, after many years of obstetric practice. As regards the late discussion at the Gynæcological Society, it may be said that it was not so judicially calm or dispassionate as that which took place at Brighton. Gynæcology, as might be expected, was much more strongly represented than obstetrics on that occasion. Now, gynæcological surgeons look at these matters from quite a different standpoint to that occupied by experienced accoucheurs. Never, perhaps, having been placed in the same position themselves, they have little sympathy with their obstetrical brethren, whose sole desire, I feel confident, is to do the best for their patients under more painful and trying circumstances than any which can fall to the lot of a surgeon. I cannot otherwise account for the following remark which fell from the lips of the president, Mr. Lawson Tait: "He was perfectly certain, from what he saw in publications, and from what he heard from the lips of men in practice, that a perfectly unjustifiable amount of child murder by craniotomy was practised." Such an expression as this may, perhaps, have been applicable to some of the ignorant "men-midwives" of former centuries; but I am confident that it is utterly undeserved by ninety-nine out of every hundred accoucheurs of the present day. It is an abuse of language to apply the term "murder" to such an operation in the hands of any conscientious practitioner: for "murder" implies *malice prepense* — a bad motive, and a deliberate intention to carry it out; both of which conditions are wanting.

There appears to be a sort of theory abroad, that a man undertakes craniotomy in a deliberate, cold-blooded manner, after reasoning with himself thus: "I know that in this case a child cannot be born alive *per vias naturales*, and that I have two alternatives before me: Am I to kill the child, to save the mother? or save the child by an operation so dangerous that it will most probably kill the mother?" And, on the principle that the mother's life is more valuable than the child's, he at once chooses the first alternative. But any one who has had much experience of craniotomy, as I unfortunately have, knows that this is quite a false view to take of the situation. In nine cases out of ten, the state of things is this: the patient has a slightly malformed pelvis, the conjugate diameter being between three and four inches, so that she is capable of having a living child under favourable circumstances: probably she is a primipara, so that the attendant is not aware of the condition of the pelvis until called in to attend her: or she is a pluripara who has given birth to one or two children that happened to be below the average size; but on this occasion the child is larger than usual, or there is an unfavourable presentation, such as the forehead to the pubis, or a brow or face, which the attendant tries in vain to alter. The head is arrested for hours in the upper strait of the pelvis, and the woman is becoming exhausted. The attendant then tries to deliver by the long forceps, or by turning, and fails. He therefore sends for further advice and help, and another practitioner is called in. After consultation, they decide that, as the woman's condition is not yet critical, they may wait longer before having recourse to extreme measures; and then, after a time, the consultant tries his hand also at the long forceps, or turning, and fails to deliver. The condition of the woman is now becoming desperate: symptoms of powerless labour have set in; the pulse is 120 between the pains, and the temperature 102° or 103°; the woman is very restless, excited, and slightly delirious; the child, if not dead already from the long-continued pressure on the head, is hovering between life and death. No time is to be lost, and nothing now remains but the dreadful alternative of craniotomy. It sacrifices the child; but it, by lessening the size of the child's head, brings quick relief to the mother.

Now, what man in his senses would, under these circumstances, perform the Cæsarean section? It is a fact universally recognised that, to give it a fair chance of success, the Cæsarean operation should be one of election, and not a *dernier ressort:* hence it is that the operation has been more successful on the Continent than in Great Britain. But in such a case as I have given above, when the soft parts have been bruised and compressed by attempts to deliver with the forceps, when there is commencing inflammation in the neck of the uterus and vagina, and when the general condition is so bad, any man who would inflict on a patient the additional shock of the Cæsarean section for the doubtful chance of saving a child would be guilty of something very like woman "murder." As an instance of a case of this kind, I will give the particulars of one which occurred to myself some years ago:

On June 30th, 1872, I received a telegram to ask me to go to Monmouth to see a lady in her first confinement. She was over thirty, and had a rather narrow pelvis. She had been in labour since June 26th, four days before, when the membranes ruptured. Mr. Norman and Mr. Wilson, of Monmouth, were in attendance. Mr. Wilson had been called in on the day before my visit, and, finding the head immovable at the brim of the pelvis, attempted to deliver with the long forceps, but without success. Mr. Norman had previously made an unsuccessful attempt to turn. I arrived about 2 p.m., June 30th. On examining, I found the head partly in the cavity of the pelvis, the scalp very puffy, and the bones overlapping a good deal. The ear could be felt, a little to the left of the pubis. The patient was rather restless and excitable. Her pulse was over 120 between the pains. She had, however, taken several full doses of opium, and inhaled a good deal of chloroform. As we all agreed that she should be delivered without delay, I applied the forceps without much difficulty. After endeavouring to extract for about two hours, in concert with the pains which were very ineffectual, I found scarcely any alteration in the position of the head. As her condition was now precarious, we all agreed, after a consultation, that craniotomy should be performed. This was accordingly done by me without much difficulty, and I extracted a large male child. The placenta was retained by hour-glass contraction of the uterus and adhesions, and I had to introduce my hand and detach it. There was some accompanying hæmorrhage. Soon after the placenta was removed the patient appeared to be in a state of great collapse; her pulse was over 130, and could scarcely be counted; her breathing was rather stertorous, and her jaw dropped. Mr. Wilson, however, was disposed to attribute this very much to the after-effects of the great quantity of chloroform and opium she had taken. But she soon rallied under stimuli, and before I left Monmouth that evening was much better. I heard afterwards that for some days she appeared likely to do well; but that about a fortnight after delivery she had severe rigors, followed by symptoms of septicæmia and pleurisy, which terminated fatally about three weeks after delivery. No one can doubt that this result was caused by the protracted labour, which lasted over five days; by the unsuccessful attempts to deliver, and by the *post-partum* hæmorrhage; but not by the craniotomy, which was neither a difficult nor a long operation. I have no manner of doubt, myself, that if the Cæsarean operation had been performed, she would have died at once from the shock.

The cases in which one has the deliberate choice, early in the labour, between craniotomy and the Cæsarean section are, according to my experience, very few and far between. It is comparatively rare to find the pelvis so much distorted that the conjugate diameter is under three inches, so that one knows at once after an examination that a living child cannot pass. The following case, however, was one of this kind:

I was sent for on Sunday, March 9th, 1856, to see a woman living in Wilder Street, who was being attended by Mr. Biggs, one of my pupils at the Bristol Medical School. When I arrived she informed me that she was confined at Birmingham with her first child sixteen months ago; that she had a very bad time; that seven doctors were in attendance, and that the child was destroyed. Mr. Biggs saw her first at half-past six the evening before: the os uteri was then but little dilated, and he could make out no presentation. At twelve the membranes ruptured, the pains became very strong: the cord then prolapsed, and he attempted to reduce it, but without success. The pains then abated in force and frequency until I saw her, about half-past four p.m.

At that time she seemed somewhat exhausted; her pulse was 130, and her pains were neither frequent nor strong. On examining, the first thing I felt was the upper part of the sacrum, which was exceedingly prominent, and also a large loop of umbilical cord quite destitute of pulsation. This was a great relief to my mind; for I then knew that there was no necessity for the Cæsarean operation. The os uteri was soft and dilatable, and somewhat œdematous and irregular in shape. The head was high above the pubis, and could only be reached with some difficulty by the forefinger of the left hand. On examining the brim of the pelvis carefully, I found that it was of an hour-glass shape, the right side being much more capacious than the left. The conjugate diameter was very narrow—in fact, not amounting to much more than two inches; for I could but just introduce the tips of the three first fingers when placed in a line between the sacrum and pubis. As the child was dead and there was nothing to be gained by further delay, I at once proceeded to perforate the head. I had some difficulty in doing this, owing to the anterior situation of the head above the pubis. After evacuating nearly all the brain, I succeeded in introducing the craniotomy forceps, and bringing away the greater portion of the skull piece by piece. The head was then drawn down into the right side of the pelvic cavity, sufficiently far for me to reach the petrous portion of the temporal bone, and to obtain a firm hold upon this part with the crotchet. Whilst thus extracting, the base of the skull suddenly came through the narrow pelvic brim with a jerk, and the rest of the delivery was accomplished without much trouble. Some hour-glass contraction of the uterus rendered it necessary to introduce the hand for the removal of the placenta. M. xxx. of laudanum, and the same quantity of sal-volatile, were given. The woman recovered without a bad symptom, although it took me four hours of hard work to deliver her. Had the child been alive, this case would undoubtedly have been a proper one for the Cæsarean section. Owing to the amount of pelvic deformity, craniotomy was rendered unusually difficult, and therefore proportionally dangerous; but yet undoubtedly safer than the Cæsarean section under the most favourable circumstances. The saving of the child's life, however, ought, under other circumstances, to have settled the matter in favour of laparotomy.

I will now briefly mention another case, in which I had not the slightest hesitation in deciding at once in favour of the Cæsarean section. In 1862 Mr. Grace, of Kingswood, brought a primipara with deformed pelvis to the Bristol General Hospital. She was a dwarf only four feet in height, and I found on measuring the pelvis that the conjugate diameter was about one inch and four-fifths in length. As the labour was just commencing, I at once decided upon not attempting delivery by craniotomy, and advised the Cæsarean section. After consulting with my colleagues, who all agreed with me, the operation was performed without delay by Mr. Coe, the senior surgeon. It was carefully and skilfully done; but the woman died within forty-eight hours. The child was saved, and is, I believe, now living. At the *post-mortem* examination, we found that the fatal result was, in all probability, due to the wide gaping wound in the uterus. But this happened before the days of antiseptic surgery, uterine sutures, or drainage tubes. The case is published in the *Obstetrical Transactions* for 1864.

The two cases just related are, however, exceptional cases: in nine cases out of ten in which craniotomy is performed, the pelvic deformity is not so great, the conjugate diameter being between three and four inches, and therefore the accoucheur has very good reason to hope that he will be able to deliver a living child by the long forceps or by turning. Indeed, he would be very blameworthy if he at once performed laparotomy without trying these methods. Accordingly he tries them, and after him others try them, but without success. In the meantime the condition of the woman goes on from bad to worse, and he is at last reduced to the hateful necessity of craniotomy. By this he sacrifices the child, but nearly always saves the mother; whereas the Cæsarean section at such a crisis would almost inevitably have killed the woman, and would, very probably, not have saved the child. Of course in all such cases it is the duty of the practitioner to induce premature labour, should he be engaged to attend the patient in a subsequent confinement.

In reading the account of the discussion which took place at the Gynæcological Society, I could not help being struck with the very exaggerated idea which seemed to prevail in the minds of many members present as to the dangers of craniotomy as an operation. Dr. Meadows, who led the discussion, is very sanguine about the success of a new method of performing the Cæsarean section, which has been introduced by Sänger of Leipzig. This method consists in stripping back the peritoneum from the edges of the uterine incision, and slicing away a portion of the muscular tissue, so that the cut edges of the peritoneum can be brought very exactly in apposition by a number of superficial sutures, in addition to the deep ones already inserted.

This method has not yet had a sufficient trial to give very reliable statistics; but, as far as it goes at present, it gives a maternal mortality of a little over 21 per cent., and a fœtal mortality of about seven. This small maternal mortality, Dr. Meadows says, shows the operation to be "little, if at all, more dangerous than craniotomy." This is a conclusion, however, from which I must entirely dissent. I have, unfortunately, as I said before, had a large experience of craniotomy, not amongst my own patients, but amongst those of others who have called me in in consultation. This will readily be understood when I mention that from 1850 to 1875 I had almost a monopoly of the consulting midwifery in this district, and consequently saw some of the worst cases which can happen. I cannot recall a single instance in which death could fairly be attributed to the operation. In those which terminated fatally, the result was due to the following causes: (1) Protracted labour. The case was attended by an ignorant midwife, who left the patient so long in labour before sending that she was almost moribund, and the infant had been dead for some hours when I arrived. The length of the labour was, no doubt, the cause of death in the Monmouth case I have above related. (2) Convulsions occurring during pregnancy. Turning was resorted to as a last resource; but the head was arrested by a narrow pelvic brim, and had to be opened. (3) Hæmorrhage from placenta prævia. Craniotomy was performed for a similar reason to that mentioned in the last case. (4) Exhaustion arising from semi-starvation, owing to long-continued vomiting for some weeks before delivery. The breech presented, but craniotomy was necessary, because the head could not be drawn through the brim of a deformed pelvis. In none of these cases was the operation particularly tedious or difficult. I therefore am led by experience to agree thoroughly with the following very sensible and judicious remarks made by Dr. Barnes, in the course of the discussion on craniotomy: "The problem would be nearer solution could we bring the Cæsarean section in some form to such perfection that the mortality attending it would be reduced to the mortality attending craniotomy. And here he disputed the validity of the statistics cited by Dr. Meadows. Dr. Meadows puts the mortality of the Cæsarean section, according to Sänger's method, at 20 per cent. Admitting this for the purpose of argument, and admitting further that the operation might be so improved as to attain an even smaller mortality, he would still most emphatically protest against the statement that the necessary mortality attending craniotomy approached 20 per cent., or even 5 per cent., excluding the cases of extreme pelvic contraction, which forbade the hope of extracting the child after craniotomy, and which all acknowledged should be treated by the Cæsarean section. Craniotomy done under fair conditions, such as are postulated for the Cæsarean section—that is, done at a chosen time with due skill—does not involve any maternal mortality."

With this last remark I thoroughly agree, and I will now proceed to answer, as well as I can, the oft-repeated question, "Ought craniotomy to be abolished?" To this I reply, Certainly not, if the child be undoubtedly dead, and there is marked disproportion between the size of the head and the capacity of the pelvis. In such cases craniotomy should always be preferred to delivery by the forceps: it is not more difficult to perform (except in those extreme cases of distortion where the Cæsarean section should be preferred), and it is much more scientific; for, by reducing the size of the child's head, it at once removes the disproportion which is the obstacle to delivery, and relieves the soft parts lining the pelvis from undue pressure. I cannot therefore agree with Dr. Meadows "that the perforator should be laid aside as an instrument of a bygone day," or that the operation *per se* is worthy to be called "an abominable, unscientific, and brutal proceeding." It is only when the child is alive that such opprobrious epithets are in any way applicable to it.

It is a remarkable circumstance that in the majority of cases in which I have had to deliver by craniotomy, I had certain proofs of the death of the child before operating. In some the cord was prolapsed and pulseless, thus showing how frequently a deformed, irregularly-shaped pelvis predisposes to this accident. In others the breech or .feet presented, or the child had been turned, and the head was arrested in the narrow pelvic brim, until it caused death by pressure on the cord; in others the child was putrid, and in others the operation had been partially completed before I arrived. In others I had no such certain proofs of the death of the infant; but there were no fœtal heart-sounds to be discovered.

It is a pity that this question, which has been so much debated of late, has not been expressed in other and more precise terms. It may reasonably be asked whether it ought to include every kind of embryotomy, such as evisceration or decapitation in arm presentations, when turning is impossible, or even tapping a hydrocephalic head—a proceeding not necessarily but usually fatal; for when the contained water is evacuated, the skull collapses under pressure, and the somewhat diffluent brain usually breaks up and comes away with the fluid.

What we all really wish to abolish is, any operation during labour which destroys the life of the child. The real question, I think, ought to be, not "Ought crainotomy to be abolished?" but, "Ought fœticide during labour to be abolished?" The law holds that there is such a thing as "justifiable homicide." Is there such a thing as "justifiable fœticide"? I believe that, under certain circumstances, there is; but it is a horrible alternative, involving an awful responsibility, such as no man should face single-handed, if possible. Could we do away with this, it would be a "consummation most devoutly to be wished!"

I fear however that, in the present state of obstetric science, there is not much chance of this. But yet we have many signs of encouragement, when we look back to the prevalent state of things forty or fifty years ago. When I began midwifery practice, craniotomy was much more common than it is now, owing to the much less frequent use of the forceps. Obstetric practitioners seemed to have a dread of the long forceps: they considered it a dangerous instrument, and confined themselves almost entirely to the short forceps. If the head remained so high that the ear could not be felt, or if the os uteri were not fully dilated, they were shy of making any attempt with the forceps, and too frequently resorted at once to craniotomy. As Dr. Routh remarked in the course of the discussion: "When we read that so eminent an obstetrician as Dr. Robert Lee had 137 perforations to 55 forceps cases, it was manifest times had greatly changed now for the better." This change has been mainly due to the late Drs. Radford and Tyler Smith, who were amongst the first to awaken the consciences of their medical brethren, by writing against this opprobrium of British midwifery; to Sir James Simpson, who improved the long forceps, and re-introduced the practice of turning as an alternative for the use of this instrument; to Dr. Barnes, who increased the length and improved the shape of the long forceps; and also to Professor Tarnier, of Paris, who invented the traction forceps, thus adding considerably to the tractive power of the instrument. Partly owing to these improvements, and partly owing to the awakened conscience of the profession, the sacrifice of the life of the child, to save that of the mother, is not a thing of common occurrence in the present day; but yet the idea that such an operation as craniotomy can be altogether done away with is at present too Utopian to be entertained, because we do not yet possess a sufficiently safe operation to substitute for it.

To answer the question, "Ought craniotomy to be abolished?" fully and finally, we ought to regard it in its relations to the three degrees of pelvic deformity which are given in all obstetric works. Firstly, in those cases in which the conjugate diameter is between three and four inches, where it is possible to deliver a living child by the forceps. In this class of cases craniotomy should not be abolished. If the child be dead, it is then to be preferred to the forceps. If it be alive, it is the only safe *dernier ressort* after the forceps or turning have been tried and have failed. Secondly, in those cases in which the conjugate diameter is between two and three inches, rendering it impossible to deliver alive a child of ordinary size at the full term. In cases of this kind, undoubtedly, craniotomy ought to be abolished. The child's life ought to be saved by laparotomy, at the least possible risk to the mother. To insure this, the Cæsarean section ought to be an operation of election, and not a last resource. It should be done at the beginning of the labour, and under the most favourable conditions. For instance, the strictest antiseptic precautions must be adopted; and in the case of the very poor, the woman should be removed, if possible, from her ordinary surroundings into some well-regulated lying-in hospital. As Dr. Routh very justly remarked in the course of the discussion at the Gynæcological Society: "In a hospital with all the necessary appliances, surgical and antiseptic, and with qualified assistants and an accustomed operator, the case was very different from what occurred in some of the slums of a town. In an ill-ventilated room, dirty, the sleeping, eating, dying room, especially if small, what were the chances of success either to mother or child? We should never forget that if abdominal surgery was more successful now than formerly, it was wholly owing to the system of cleanliness and antiseptics employed. When a man was suddenly called to an urgent case at night in one of these slums, surely craniotomy offered the best chance to the mother." Cases of this kind are pre-eminently suited for the induction of premature labour: if the accoucheur has the good fortune to see his patient, and make a careful examination of her pelvis a few months before delivery, he may thus avoid the dreadful responsibility of having to choose between craniotomy and the Cæsarean section. Thirdly and lastly, craniotomy ought to be abolished in those cases where the conjugate diameter is under two inches. It is true that even in such cases it is sometimes possible to deliver a mutilated child by the cephalotribe, or even by craniotomy; but the operation is so difficult and dangerous to the mother, if it succeeds, that the risk she would incur would be almost, if not quite, as great as that of the Cæsarean section; and if it fails, the Cæsarean section would have to be done under the most unfavourable conditions. There should be no hesitation, then, in at once adopting the latter operation, and thus saving, if possible, the lives of both mother and child.

